# *Rinegan*: A Scalable Image Processing Architecture for Large Scale Surveillance Applications

**DOI:** 10.3389/fnbot.2021.648101

**Published:** 2021-08-23

**Authors:** Xi Luo, Lei Feng, Hao Xun, Yuanfei Zhang, Yixin Li, Lihua Yin

**Affiliations:** ^1^Cyber Space Institute of Advanced Technology, Guangzhou University, Guangzhou, China; ^2^School of Cyber Security, University of Chinese Academy of Sciences, Beijing, China

**Keywords:** smart gateway, large scale, image processing, intelligent security robot, microservice

## Abstract

Image processing is widely used in intelligent robots, significantly improving the surveillance capabilities of smart buildings, industrial parks, and border ports. However, relying on the camera installed in a single robot is not enough since it only provides a narrow field of view as well as limited processing performance. Specially, a target person such as the suspect may appear anywhere and tracking the suspect in such a large-scale scene requires cooperation between fixed cameras and patrol robots. This induces a significant surge in demand for data, computing resources, as well as networking infrastructures. In this work, we develop a scalable architecture to optimize image processing efficacy and response rate for visual ability. In this architecture, the lightweight pre-process and object detection functions are deployed on the gateway-side to minimize the bandwidth consumption. Cloud-side servers receive solely the recognized data rather than entire image or video streams to identify specific suspect. Then the cloud-side sends the information to the robot, and the robot completes the corresponding tracking task. All these functions are implemented and orchestrated based on micro-service architecture to improve the flexibility. We implement a prototype system, called *Rinegan*, and evaluate it in an in-lab testing environment. The result shows that *Rinegan* is able to improve the effectiveness and efficacy of image processing.

## 1. Introduction

Image process has been widely implemented in intelligent robots, which significantly improve the visual ability of smart buildings, industrial parks, border ports and so forth. For example, patrol robots, a critical partner of police officers or area administrators for security surveillance, are usually equipped with cameras that can track suspicious persons by collecting and processing images or video streaming. However, such track task solely relying on individual and narrow view of a single robot is no longer effectiveness in large scale environment. Specifically, the suspect may appear anywhere, and the coverage of a single robot is limited, which is not enough to deal with this situation. Hence, the patrol robots have to cooperate with surveillance cameras distributed in such monitored area. When a suspect appears somewhere, once the surveillance camera captures him, it will send the information to a nearby patrol robot through a communication channel, and the robot will come and track the target person in time.

Such a large scale cooperation of robots and cameras induces a significant surge in demand for image or video data collection, computing resources for object detection and suspect recognition, as well as communication bandwidth of networking infrastructures. A number of researches (Li et al., 2015; Cai et al., 2016; Redmon et al., 2016; Shi et al., 2018; Tan et al., 2020) have dedicated to improve the ability in object detection by employing supervised learning or reinforcement learning method. These works consider only the visual information collected from the camera implemented inside robot, which are confined in a small surveillance area. Hence, some researchers, e.g., Dolberg et al. (2016), Meng et al. (2017), Chen et al. (2018), Lee et al. (2018), Bevi et al. (2019), Bistritz and Bambos (2019), Chang et al. (2019), and Li et al. (2019), focus on designing edge computing architecture to achieve a high level of scalability and fast response rate, comparing with the widely deployed centralized cloud-based(IoT) solutions. Some others, e.g., Kovatsch et al. (2012), Morabito and Beijar (2016), Morabito et al. (2016, 2017), Morabito (2017), Rufino et al. (2017), Cheng et al. (2018), Dolui and Király (2018), Mendki (2018), and Ogawa et al. (2019), also works on the virtualization of edge-side gateways to enhance the flexibility of above edge computing architecture. All these works either improve the communication and computation efficacy at infrastructure level or design an edge-cloud cooperation training mechanism in which training task runs on cloud side while detection and recognition tasks are executed on the edge. However, they neglect the advantage of the decoupling between the object detection and suspect recognition. Specifically, a patrol robot requires only the location and suspect information rather than the entire image or video stream. Therefore, only the required data are transferred to save the bandwidth. A camera with limited resources can only run an object detection process and send result to cloud side for recognition, and the robots receive only the location and suspect information for tracking process. In this case, both the resource consumption and the risk of data leakage caused by network communication are minimized.

In this work, we present a scalable edge-cloud cooperation architecture, which harmonizes the object detection and recognition applications to facilitate the image processing ability of intelligent robots. In our architecture, the image processing is separate into four phases, i.e., pre-process, object detection, representation, and recognition. The pre-process and object detection tasks are deployed on the edge-side gateway to minimize the response delay and the bandwidth consumption. The cloud-side obtains and processes solely the detected objects from the edge-side smart gateways. All these functions are orchestrated using micro-service techniques, which provide a high level of modularity and interoperability, to optimize the resource allocation. As a result, a robot receives only the location and suspect information for tracking process. Facilitated by the proposed architecture, the robots are able to effectively surveil the entire environment and therefore can make accurate response than that rely on their inside cameras. We implement a prototype system, called *Rinegan*, and evaluate it in an in-lab testing environment. The result shows the efficacy and effectiveness.

In summary, the contributions of our work are as follows.

1. We propose a hierarchical architecture to enhance the efficacy of object recognition applications. The lightweight object detection functions are assigned distributively on gateways to minimize bandwidth consumption between edge and cloud. Therefore, the cloud-side servers receive only structural objects and the recognition result is send to the nearby robots. This architecture greatly reduces the load pressure and computation resources and improves the tracking ability of robots.
2. We develop a prototype system, i.e., *Rinegan*, by implementing the object functions into micro-service instances. Therefore, *Rinegan* can reasonably and deftly orchestrate the tasks like object detection and recognition. In addition, the isolated property in this system can also helps to protect the sensitive information reserved in video data (Wang et al., 2020), i.e., only the required data is transferred through network.
3. We deploy *Rinegan* in a in-lab environment to evaluate its performance. The result shows that *Rinegan* achieves outstanding data processing efficacy and excellent scalability comparing to traditional centralized mode. We have reason to believe that Rinegan can also be applied to large-scale scenarios like smart building (Qiu et al., 2019).

### 1.1. Organization

The rest of the paper is organized as follows. In section 2, we illustrate the background and motivation, as well as the related work. In section 3, we provide the designation of our proposed gateway system. In section 4, we conduct experiments in a in-lab environment and make comparative analysis and evaluation of its performance. In section 5, we discuss the shortcomings of this work and looked forward to the follow-up work. At last, in section 6, we conclude our work.

## 2. Background and Motivation

When human beings build the world around us, we often expect to make copies like ours, which can better adapt to the environment and habits in human life. Studies have found that a certain area of the human brain is specifically used to recognize things and make cognitive responses. As a result, people also hope to implement a function such as face recognition on a intelligent security robot to help humans work. In this section, we briefly introduce the background of intelligent security robot, image processing, gateway virtualization and micro-service architecture to illustrate the motivation of our work.

### 2.1. Related Work

Regarding target detection and recognition, many people are committed to using cloud computing to achieve it. Yaseen et al. (2018) proposed a video analysis system based on cloud computing to realize automatic analysis of video stream data. The experimental results show that the system can be expanded to a certain extent according to the number and size of video streams. However, the author did not consider the load capacity of the cloud in large-scale scenarios. When the amount of data that the system needs to process is particularly large, the cloud may be overloaded, and we can use edge computing to solve this problem. Qi et al. (2017) combined smart phones and cloud computing to implement a DNN-based target detection system, which is mainly used in vehicle detection and recognition. We noticed that in their work, the authors use smartphones as a video stream acquisition tool. Tasks such as image processing and object detection are processed in the cloud. Such a distributed system may not be suitable for processing a large number of images and videos on congested roads. Our system is implemented through microservices, which can schedule edge-side gateways to process images, taking into account both distributed and large-scale scalability.

Using cloud computing alone is not enough to deal with large-scale scenarios. Some people consider reducing the pressure on the cloud at the edge. Sun et al. (Tian et al., 2019) points out that edge computing can share the load of the cloud center and provide a service environment and computing capabilities at the edge of the network. Moreover, edge computing has low latency and higher bandwidth. Ren et al. (2018) combined edge computing, but they use servers on the edge side, which is difficult to deploy. Moreover, the cloud is only responsible for training the model, and does not use microservice technology to schedule image processing tasks. And Tian et al. (2020) and Luo et al. (2020) mentioned that although cloud computing and cloud storage have brought great convenience, the security issues cannot be ignored. The distributed attack detection system deployed on edge devices in this article also supplements the deficiencies of cloud-only.

About the architecture of edge computing, Cha et al. (2018) designed a blockchain-based smart gateway solution, which uses a digital signature mechanism to effectively protect privacy, and at the same time, it can adaptively maintain user privacy of devices in the network. Mouradian et al. (2018), referred to the ideas of network function virtualization and SDN, and propose a distributed and dynamic configuration gateway structure to solve large-scale disaster management applications. Constant et al. (2017), developed an intelligent gateway system based on fog computing. This architecture can use a knowledge-based model to enhance the quality of interaction between wearable IoT devices and the cloud. Rahmani et al. (2018), used the key position of the gateway at the edge to provide higher-level services, and propose a smart electronic health gateway for smart medical care. We use the concept of fog computing to propose a fog-assisted system architecture that can cope with many of the medical systems challenge. Li et al. (2016), proposed an SDN-based architecture for solving development-level IoT solutions, making devices and gateways programmable for developers, which can quickly reuse ready-made programs and data to create IoT applications. However, none of the above articles takes into account the scalability of the architecture. We use microservice technology to deploy and schedule tasks for edge devices more flexibly to adapt to large-scale scenarios.

### 2.2. Layout of Intelligent Security Robot Applications

**Figure 1:**
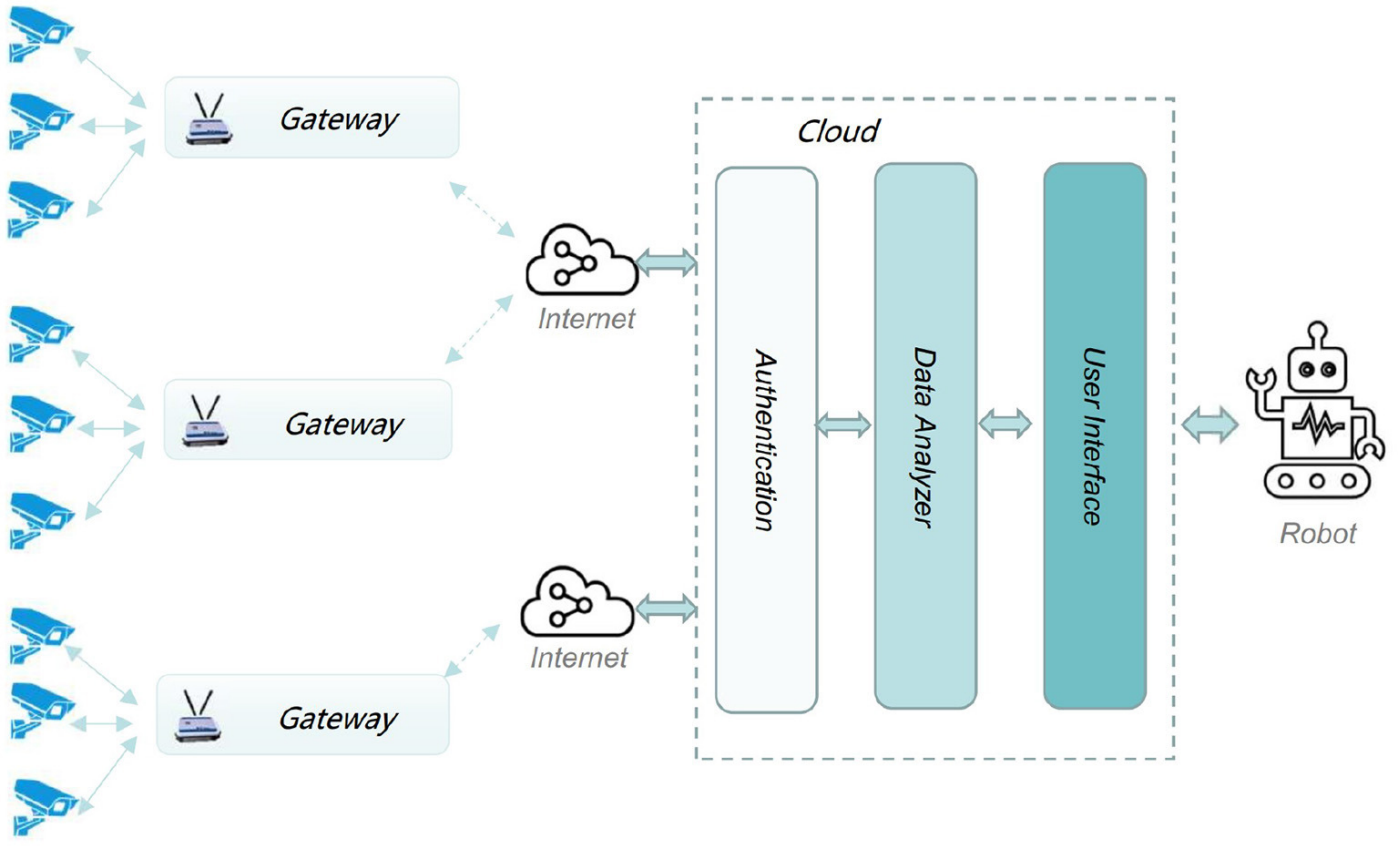
Layout of intelligent security robot applications.

Figure 1 plots the common architecture of intelligent security robot applications. Devices, e.g., security cameras, connect to gateway. Such a gateway provides Internet accessibility for a set of devices. In general, the robot involves functionalities like authentication, data analysis and user interface.

Basically, the gateway should have capability of discovering devices when user makes a “Discover” request. Then, polling of discovered devices should be initiated once the manage platform makes a “Approve” request. The gateway should also make a necessary control action and return “Success” or “Failure” response whenever it receives a control request from the intelligent security robot.

However, the ever-increasing large scale data streams significantly challenge the bandwidth and computation resources of the centralized intelligent security robot infrastructure. For instance, image data collected by the cameras in a smart city application will exhaust the communication bandwidth between gateway and intelligent security robot and disrupt the computation ability of robot-side.

### 2.3. Image Processing

**Figure 2:**
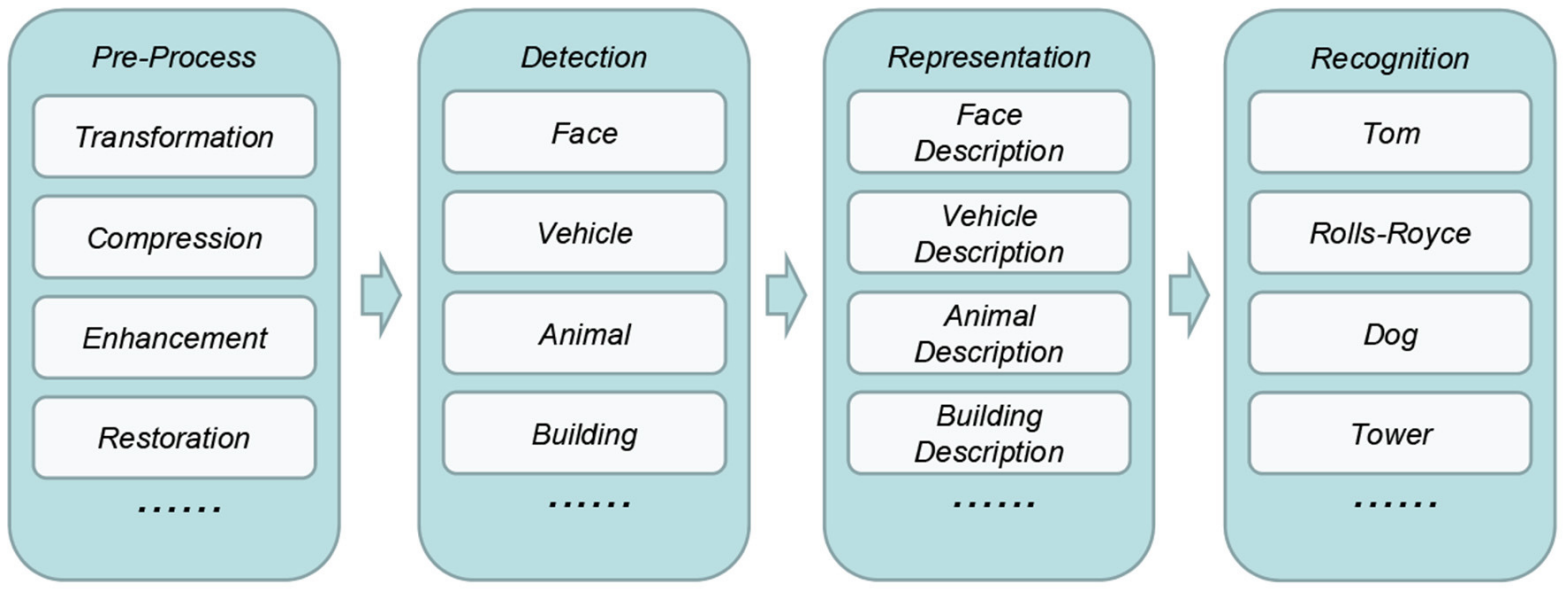
Workflow of image processing.

In the object recognition function of nowadays intelligent security robot, image processing is one of the most important functionality. It enables visibility for these things and applications. Figure 2 briefly illustrates the workflow of it, which consists of four phases, i.e., pre-process, object detection, representation, and recognition.

In pre-process phase, the raw image data will be transformed, compressed, enhanced, restored and so forth to improve its quality and facilitate the following process. In detection phase, specific objects involved in will be detected and extracted by using corresponding models such as face, vehicle, animal, and building models. In representation phase, these objects will be described as a set of features. Finally, in recognition phase, these objects will be classified as specific person, car, animal, or building.

In general, above four phases are treated as a whole when deploying into intelligent security robot system. Specifically, all of them are either deployed on the edge-side devices or on the cloud-side servers. Both scenarios are not scalable enough to large scale environment. On one hand, most of edge-side devices, even the gateways, are resource-constraint, which are not capable of executing all these tasks. On the other hand, cloud-side deployment significantly increase the stress of such centralized architecture.

### 2.4. Gateway Virtualization

The possibility of introducing lightweight virtualization technologies, and in particular container-based virtualization, in this kind of environments allows having a system that benefits of the main features introduced by containers, if compared to alternative solutions such as hypervisor-based virtualization or hybrid solutions (Chang et al., 2019): (i) Fast building process, instantiation and initialization of containers. (ii) High density of application/services due to the small container images.

**Figure 3:**
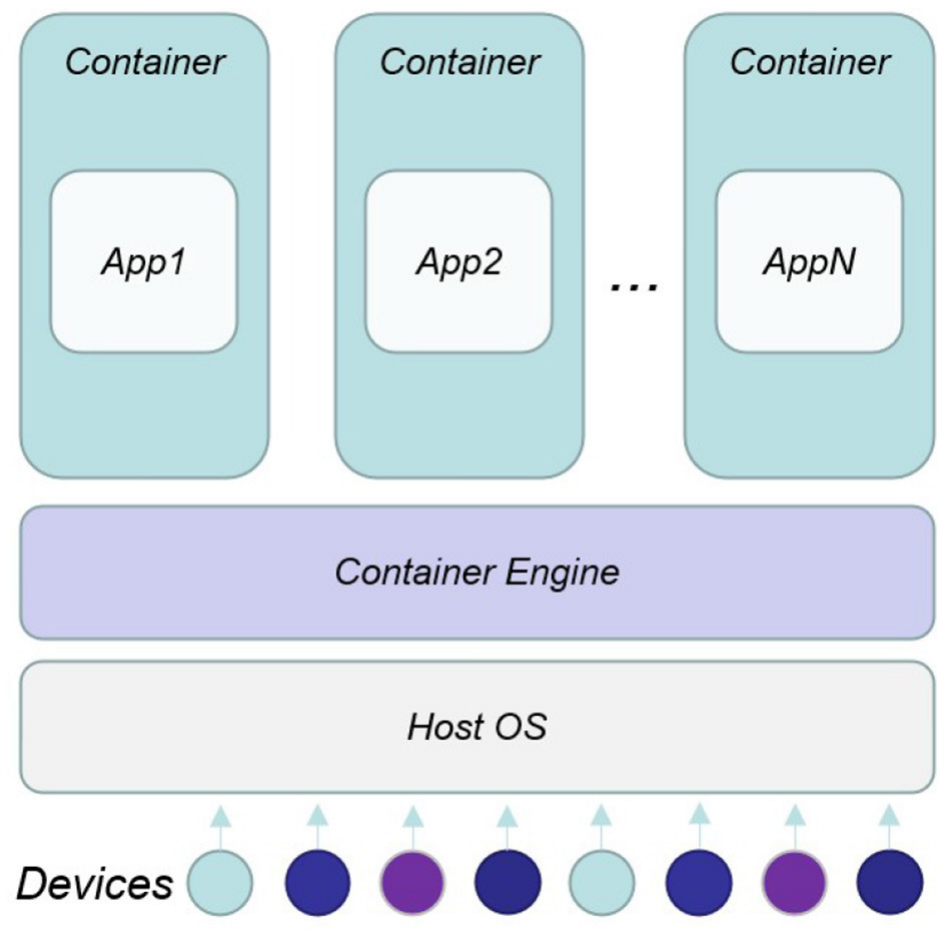
Architecture of containerized gateway.

Container-based virtualization (Figure 3) provides a different level of abstraction in terms of virtualization and isolation compared to hypervisors. Hypervisors virtualize hardware and device drivers, which generates overhead. On the contrary, containers implement isolation of processes at the operating system level, thus, avoiding such overhead (Chang et al., 2019). Due to the shared kernel (as well as operating system libraries), an advantage of container-based solutions is that they can achieve a higher density of virtualized instances, and disk images are smaller compared to hypervisor-based solutions. Moreover, an application can be designed to work in multiple containers, which can interact each other by mean of linking system, with a guarantee of no conflicts with other application containers running on the same machine. It is exactly these features that make possible the integration of the functionality of containers in a wide range of contexts: smart devices, the intelligent security robot, and embedded systems.

### 2.5. Micro-Service Architecture

Micro-services are an architectural and organizational approach to software development where software is composed of small independent services that communicate over well-defined APIs. These services are owned by small, self-contained teams. Micro-services architectures make applications easier to scale and faster to develop, enabling innovation and accelerating time-to-market for new features.

**Figure 4:**
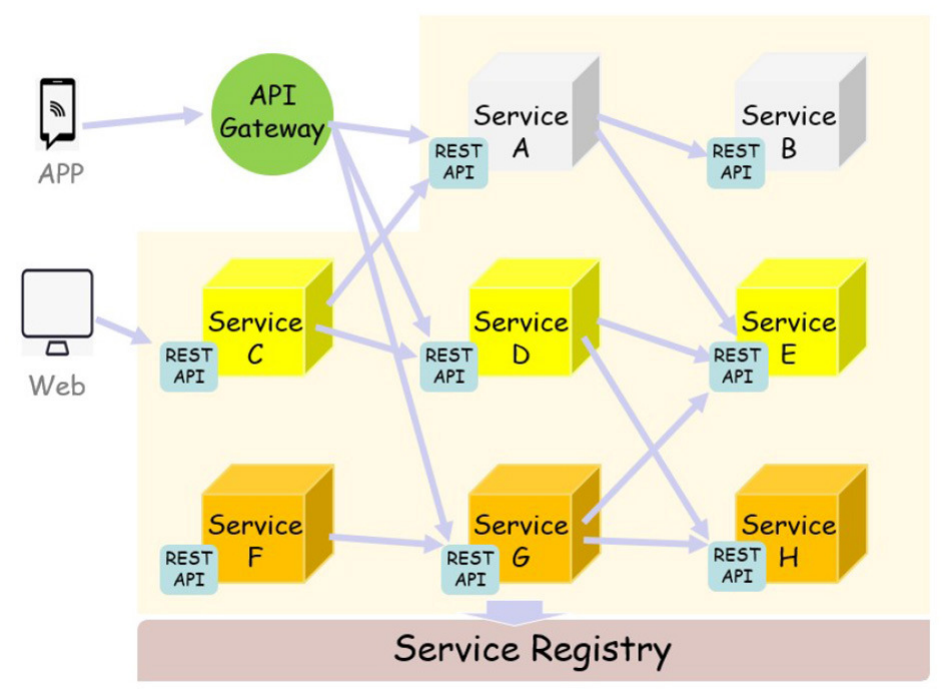
Micro-service architecture.

As shown in Figure 4, each component service in a micro-services architecture can be developed, deployed, operated, and scaled without affecting the functioning of other services. Services do not need to share any of their code or implementation with other services. Any communication between individual components happens via well-defined APIs. Each service is designed for a set of capabilities and focuses on solving a specific problem. If developers contribute more code to a service over time and the service becomes complex, it can be broken into smaller services.

Micro-services allow each service to be independently scaled to meet demand for the application feature it supports. This enables teams to right-size infrastructure needs, accurately measure the cost of a feature, and maintain availability if a service experiences a spike in demand. Micro-services enable continuous integration and continuous delivery, making it easy to try out new ideas and to roll back if something doesn't work. The low cost of failure enables experimentation, makes it easier to update code, and accelerates time-to-market for new features. Service independence increases an application's resistance to failure. In a monolithic architecture, if a single component fails, it can cause the entire application to fail. With micro-services, applications handle total service failure by degrading functionality, and not crashing the entire application.

## 3. System Design

The image processing architecture we proposed can optimize the object recognition capabilities of the intelligent security robot and improve the efficiency of image processing. We developed a system *Rinegan* based on this architecture. In this section, we will introduce this system in detail.

### 3.1. System Overview

The *Rinegan* system can effectively improve the image processing performance of the intelligent security robot in a large-scale environment. It allocates part of the image processing task flow to the gateway on the edge-side, allowing the gateway and the cloud to work together to complete the entire image processing flow. The system relieves the pressure of data transmission in the cloud, while also solving the problem of insufficient computing on the edge side. In order to achieve these effects, we have used micro-service technology to develop *Rinegan*, which has good flexibility and scalability. The reason why we use micro-services is because micro-services have a high degree of modularity and interoperability, which can improve the flexibility of the system and optimize the resource allocation of the system. In this section, we will introduce two aspects of system architecture and workflow.

#### 3.1.1. System Architecture

**Figure 5:**
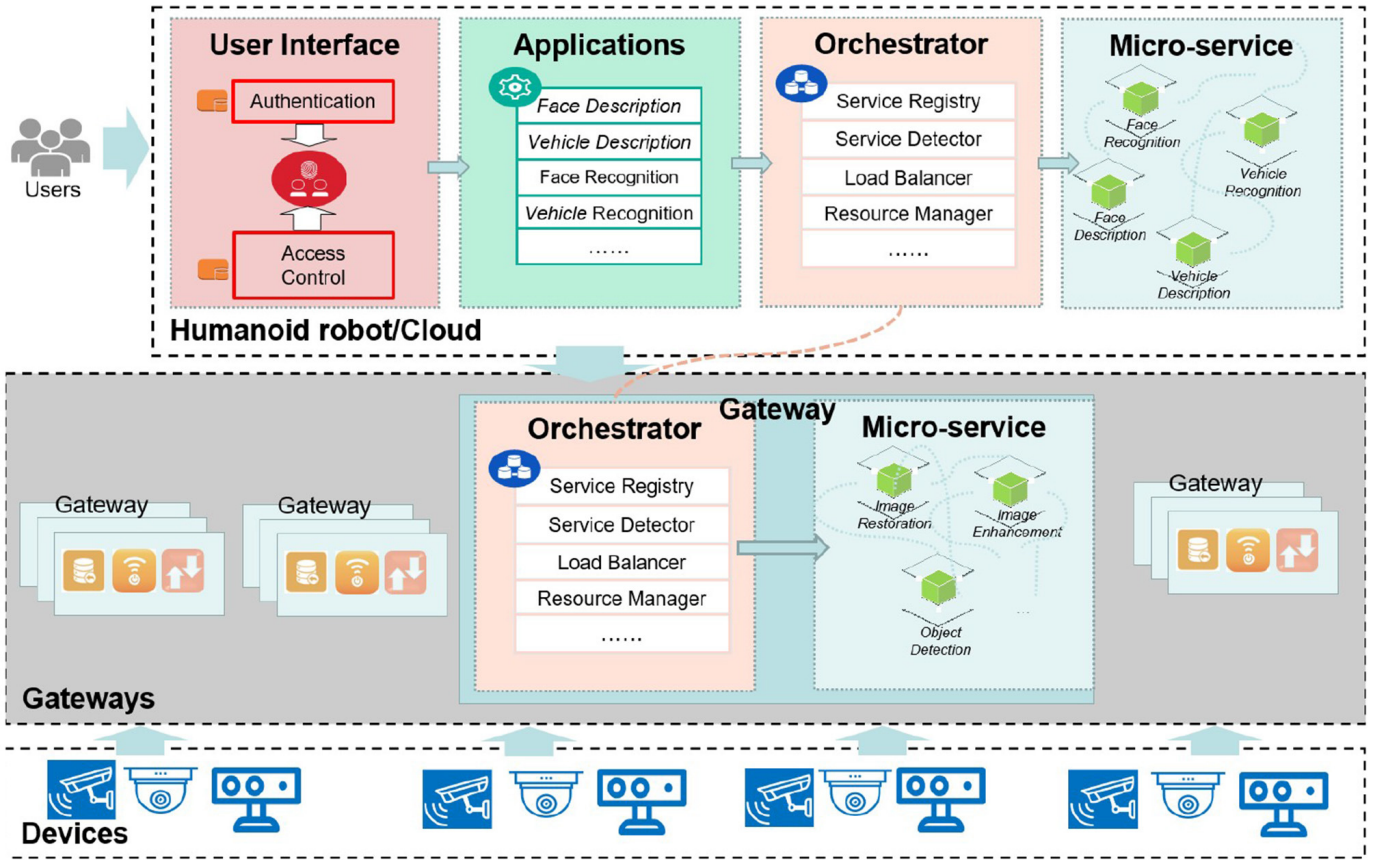
Architecture of Rinegan.

As presented in Figure 5, inspired by the structure of the IoT system, *Rinegan* can also be divided into the following three layers: *perception layer, network layer*, and *application layer*. Each layer corresponds to a kind of entity, and each layer has its own functions and services. Below we introduce the specific situation of these three layers, respectively.

1. ***Perception layer*** takes the terminal *devices* as the core, the most common ones are various sensors, in this work, specifically the cameras. The perception layer is the data foundation of the whole system, and it's also a key part of information collection. The cameras collect raw data including images and videos, then upload these data to the next layer—the smart gateway.
2. ***Network layer*** is also called the transport layer, in this work, since we are studying a scalable architecture for image processing, it can be interpreted as “*gateway* layer” in a narrow sense here. In this layer, the gateway is responsible for simple processing of images and video streams uploaded by the perception layer to reduce the amount of data transmission. Specifically, simple processing refers to prepocess images and the process of object detection, such as image enhancement, image restoration, We deployed micro-services in the gateways and made reasonable orchestration components for these micro-service. The orchestrator component contains functions like service registry, service detector, load balancer and resource manager to ensure the normal and orderly operation of micro-services.
3. ***Application layer*** can be understood as cloud server in the intelligent security robot, in this work, this layer contains four parts, including *user interface, applications, orchestrator, micro-service*. *User interface* is divided into two modules:Authentication and Access control. The authentication module is based on the identity of each user, adopts a standardized identity authentication format, and follows a certain security mechanism to ensure that the access user is a safe user. The access control module authorizes the authenticated user through the control strategy to ensure the legal use of the information resources by the user within the authorized scope. *Applications* are user-oriented tasks, when the cloud receives the image processed by the gateway, this module is responsible for object description and recognition, such as human tracking, traffic flow detection, traffic violation identification and so on. *Orchestrator* module is responsible for the orderly organization of micro-services deployed in the huamnoid robot cloud. The orchestration process is mainly implemented in the cloud server, and there a few scheduling processes in the gateway, as mentioned in the *gateway* layer. *Micro-service* is a small granular service that can be independently developed and deployed. Micro-services generally perform specific functions according to the orchestration of the *Orchestrator*, such as face recognition, face description, vehicle recognition, and vehicle description.

#### 3.1.2. System Workflow

Above we introduced the three-layer architecture of the system we designed, as well as the composition and function of each layer. In this part, we introduce how *Rinegan* works.

**Figure 6:**
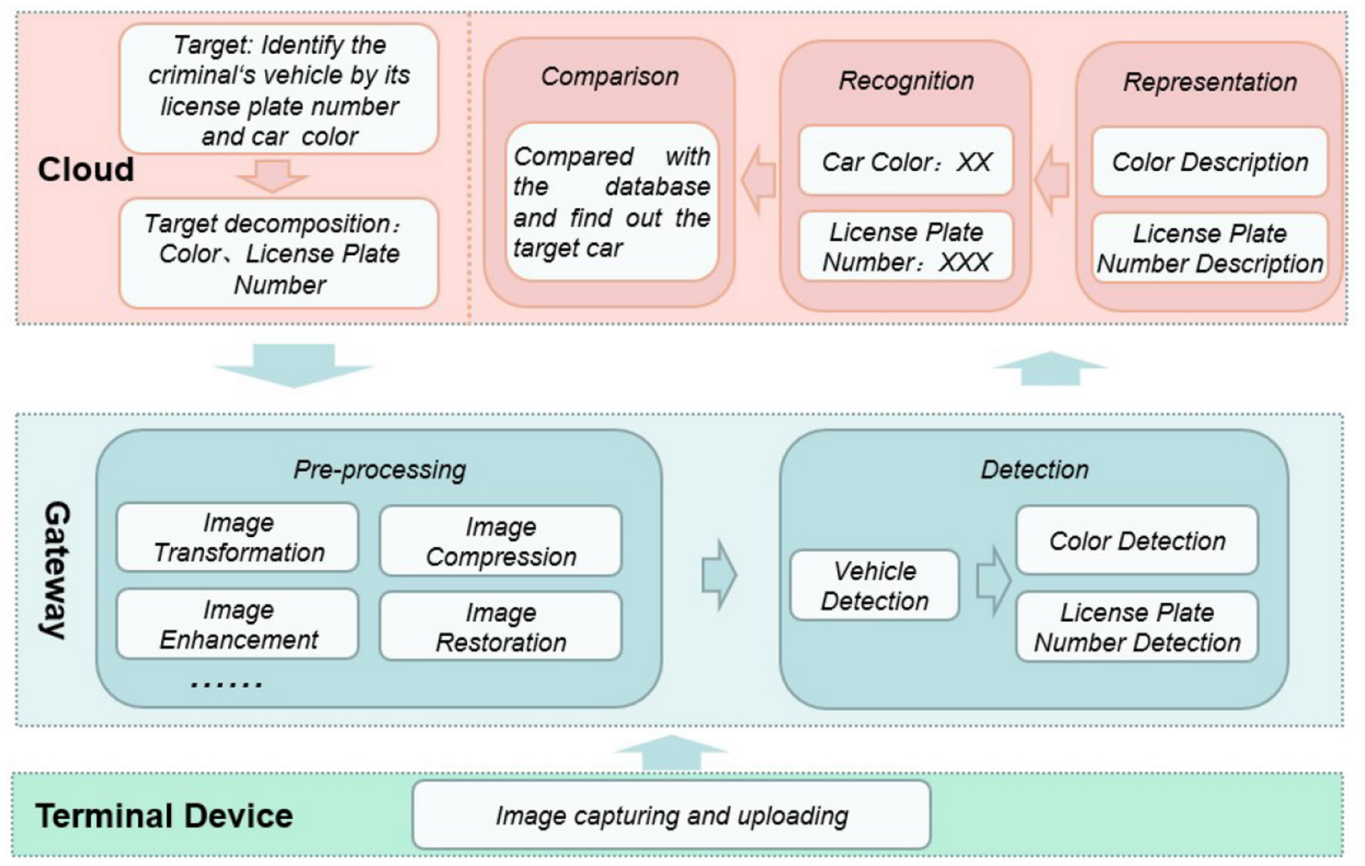
Workflow of *Rinegan*.

As shown in the Figure 6, we assume an application scenario to make the workflow more specific. Imaging a scenario where a criminal is driving a car to escape in the city. We need to make the intelligent security robot recognize the vehicle color and license plate number of the vehicle to lock its position.

The terminal camera at the perception layer can collect and store large-scale image data, which we call original images, and the camera transmits the collected data to the gateway layer. After the gateway on the edge-side receives these raw images, the image processing micro-services deployed in them start to work. The first step is *image pre-processing*. Specifically, image transformation includes grayscale and geometric transformation to reduce the amount of data that needs to be processed. Image enhancement means to enhance the useful information in the image. It can be a distorted process. Its purpose is to improve the visual effect of the image. For the application of a given image, it purposefully emphasizes the overall or local characteristics of the image. Image compression refers to the process of reducing image storage or reducing image bandwidth. Image restoration is an objective process that attempts to restore the original content or quality of images with reduced or distorted quality.

The gateway can perform object detection on the pre-processed image. The object detection is roughly divided into three steps. For this scenario, the first is the classification operation. In a given image or a video, the gateway must determine what type of target is in it, that is, the target vehicle. Then there is the positioning operation, to locate the location of the target vehicle. Finally, the detection operation is to detect the color and license plate of the targeted vehicle.

The gateway uploads the processed image to the cloud of the intelligent security robot, and executes the feature representation in the cloud. Through the feature extraction algorithm, the micro-service describes the license plate number and color of the target vehicle for identification. After the cloud recognizes the color and license plate number, compares and matches with all vehicle feature information in the database, and then the target vehicle is identified.

### 3.2. System Description

In this part, we give a detailed description of each module in the three-layer architecture of the system.

#### 3.2.1. User Interface

For a system, security is the primary consideration in the design process. To ensure the security of the system, it is necessary to ensure that the users interacting with the system are not malicious. There are two aspects of user security that need to be considered. One is the authentication of the user's identity, and the other is the acquisition of the user's operating authority to the system. In response to these two problems, we have developed two functions in the User interface, identity authentication and access control.

We have realized the authentication of the user's identity through the digital certificate. The digital certificate contains the user's identity information and digital signature, which can prove their identity to the entities in the system. The signature certificate in the digital certificate is used to sign user information. To ensure the non-repudiation of information, the encryption certificate is mainly used to encrypt information transmitted by users to ensure the authenticity and integrity of the information. Access control means that after the system completes the identification of the user, it determines the access request authority to the information resource according to the user's identity. We use the discretionary access control strategy to allow legitimate users to access the objects allowed by the policy as users or user groups, and at the same time prevent illegal users from accessing.

#### 3.2.2. Application

This module is user-oriented. After the system completes the user's identity authentication and grants the user certain access rights, the user initiates the corresponding function request to the system according to his needs, so the function of this module is specific. For example, the user may need our system to do face recognition, track a person, recognize a vehicle, etc., then this module will send these specific function requests to the server, and call the micro-service interface to complete these tasks.

#### 3.2.3. Orchestration

When a system adopts the micro-service architecture, the original business may not change, but the system has been split into many new micro-services. Compared with the traditional architecture, the micro-service architecture will rely more on the collaboration between the micro-services to achieve a complete business process. This collaboration is the service orchestration, which requires a complete orchestration framework to support.

Orchestration is oriented toward executable processes, an executable process is used to coordinate internal and external service interactions, and the overall goal, involved operations, and service call sequence are controlled through the process. The advantage of the orchestrator is that the process control service always knows where each business is going, and monitoring the business has become a relatively simple matter.

#### 3.2.4. Micro-Service

The reason why we use micro-services is because micro-services have a high degree of modularity and interoperability, which can improve the flexibility of the system and optimize the resource allocation of the system. Whether it is the lightweight image processing tasks that we deploy on the edge (e.g., image preprocessing and target detection), or the subsequent image processing tasks that we deploy in the cloud, they are all based on the corresponding micro-service module.

We have used virtualization technology and built these micro-services using Spring boot in the docker container. To ensure that the micro-services in the container are running properly, we set a module named *monitor&protection*, which has three functions: link tracking, service fusing, and service monitor. Service fusing provides a proxy for micro-services. It implements fault tolerance and self-protection by setting timeout and circuit breaker modes for network requests to prevent cascading failure when the service is impossible, which leads to an avalanche effect. Link tracking is used in the local area network to update the connection information. This function can ensure the real-time performance of the micro-service status. Service monitor is used to monitor the running status of each micro-service. When a micro-service has a problem, it will report to the server. It works with link tracking to ensure the normal operation of the micro-service in the container.

For the configuration management and service discovery of micro-services, we also designed a module called *discovery&config*. *Discovery* is RESTful-based service discovery functions, which are mainly used for the discovery and registration of micro-services. When a new micro-service is deployed, this function is responsible for registering the micro-service with the server, which ensures high availability, flexibility, and scalability through heartbeat checking, client-side caching, and other mechanisms. *Config* is used to uniformly manage the configuration of micro-services. Unlike traditional monolithic applications, in293the micro-service architecture, an application system using the micro-service architecture may contain294hundreds of micro-services. Therefore, centralized management configuration is necessary.

**Figure 7:**
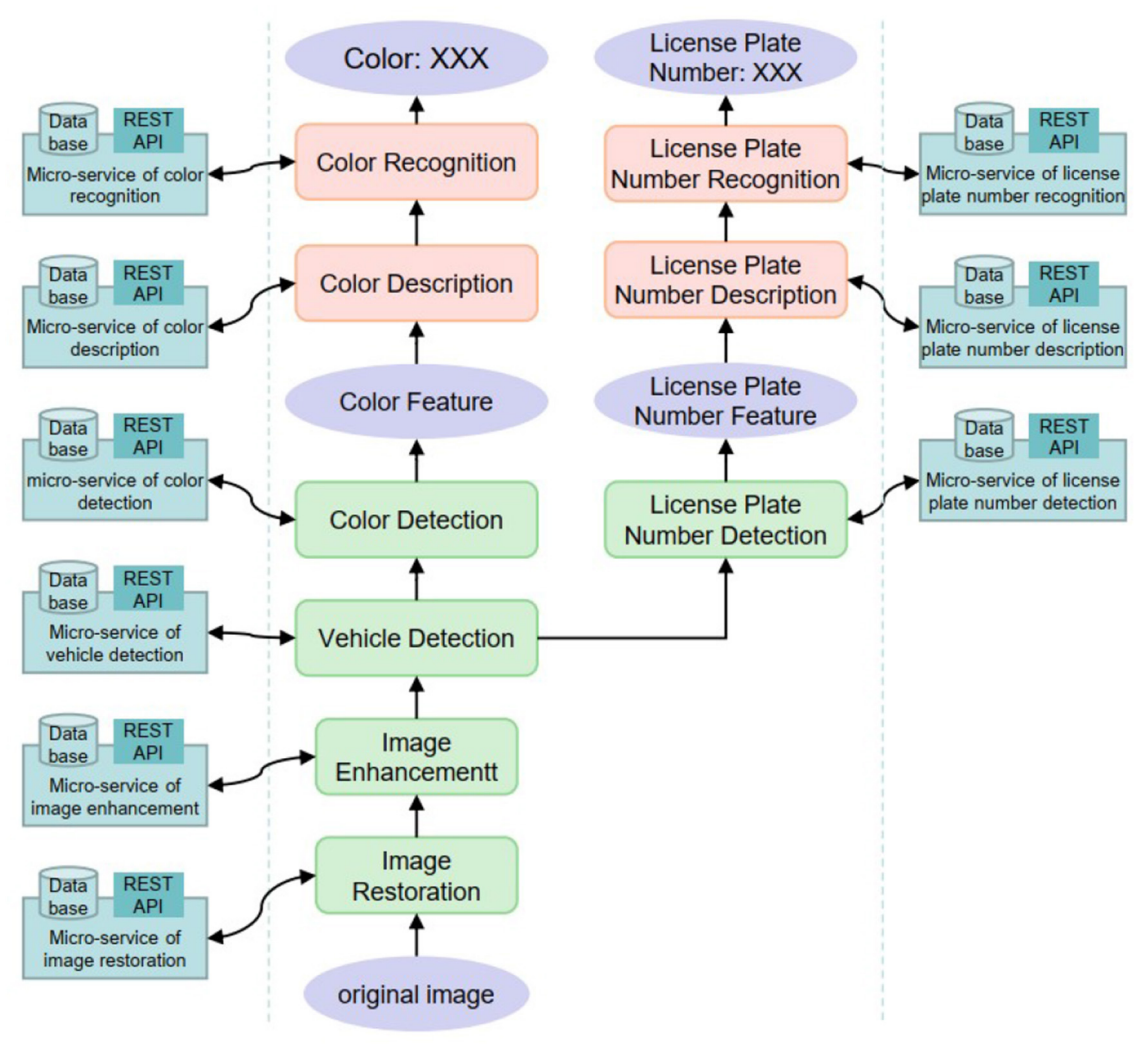
Micro-services in image processing.

As shown in the Figure 7, we not only deploy micro-services in the cloud, but also the gateway on the edge side uses the micro-services developed by us when processing images. The entire working process of the system involves the participation of micro-services from beginning to end. For example, at the gateway layer, the gateway completes image restoration, image enhancement and object detection by calling the RESTAPI of the corresponding micro-service. After the cloud receives the data processed by the gateway, the micro-services continue to perform feature description and feature recognition operations on the image, and the process of calling the micro-services is basically the same as the gateway layer.

#### 3.2.5. Gateway

In our proposed architecture and developed system, the gateway is a device on the edge-side that cooperates with the cloud to complete image processing tasks in a large-scale environment.

In addition to its own original functions, for the flexibility of the system, and to reduce the consumption of the cloud to improve the efficiency of the system, we deployed the first two steps of the image processing process in the gateway for execution, namely image pre-processing and object detection.

Similar to the cloud, we also use the docker container-based virtualization technology to deploy the corresponding micro-services, which greatly enhances the scalability of the entire system, and the orchestration and details of the micro-services are basically the same as those of the cloud.

#### 3.2.6. Terminal Devices

In this work, the terminal device is the camera, the physical device of the perception layer. These cameras collect or capture images in specific places and store them in its memory, but the cameras do not have the function of processing images, so these images are transmitted to the gateway for further screening and processing.

## 4. Evaluation

In this section, we present our experiment to evaluate the performance of *Rinegan*. We first introduce the implementation detail and then analyze the result.

### 4.1. Implementation

In order to verify that the architecture we proposed can effectively enhance the efficacy of object recognition, we implemented the protype system based on our proposed image processing architecture—*Rinegan* in our laboratory environment. According to related research (Nefian and Hayes, 1998; Lal et al., 2018), face recognition is currently one of the most commonly used function, such as tracking suspects in cities. So we choose the face recognition function to test the performance of our architecture. Note that, *Rinegan* can not only facilitate the face recognition task but also the other recognition applications such as car and animal recognition. This because these applications are all based on the image process ability just the same as face recognition.

We select 1,000 pictures (7.8MB in total) collected from “Large-scale CelebFaces Attributes (CelebA) Dataset”^1^ as our experimental data. Our purpose is to find a random select specific target person in these pictures. The face recognition and detection algorithms used in this work are “Face-recognition” project.^2^ This project provides convenient APIs, i.e., the “face_location”^2^ and “face_distance”^2^ APIs, for face detection and recognition, respectively. Moreover, we collect a MP4 video file, named “hamilton_clip.mp4”(640*360, 29.97 fps, 4.9 MB, 78 s),^3^ for evaluation. Indeed, the process of video data is not different from that of pictures except the loading task which converts the video stream to a set of frames, i.e., pictures.

In the process of image processing, we deploy the task of object detection on the edge side in which the devices detect the object in the input pictures or video, that is, the person. Finally, the picture will be cropped, and other parts except the face will be cropped off, and the edges will be sent to the cloud to recognize the face. Therefore, the cloud server only needs to do the recognition task. It is worth noting that we ignored the process of sending information from the cloud to the robot since it is a simple task which transfers only the location received from corresponding gateways and the suspect information such as name, passport ID and so forth. At the same time, we also deployed object detection and face recognition tasks all in the cloud (we call it *centralized* later), as a comparative experiment of our architecture.

**Figure 8:**
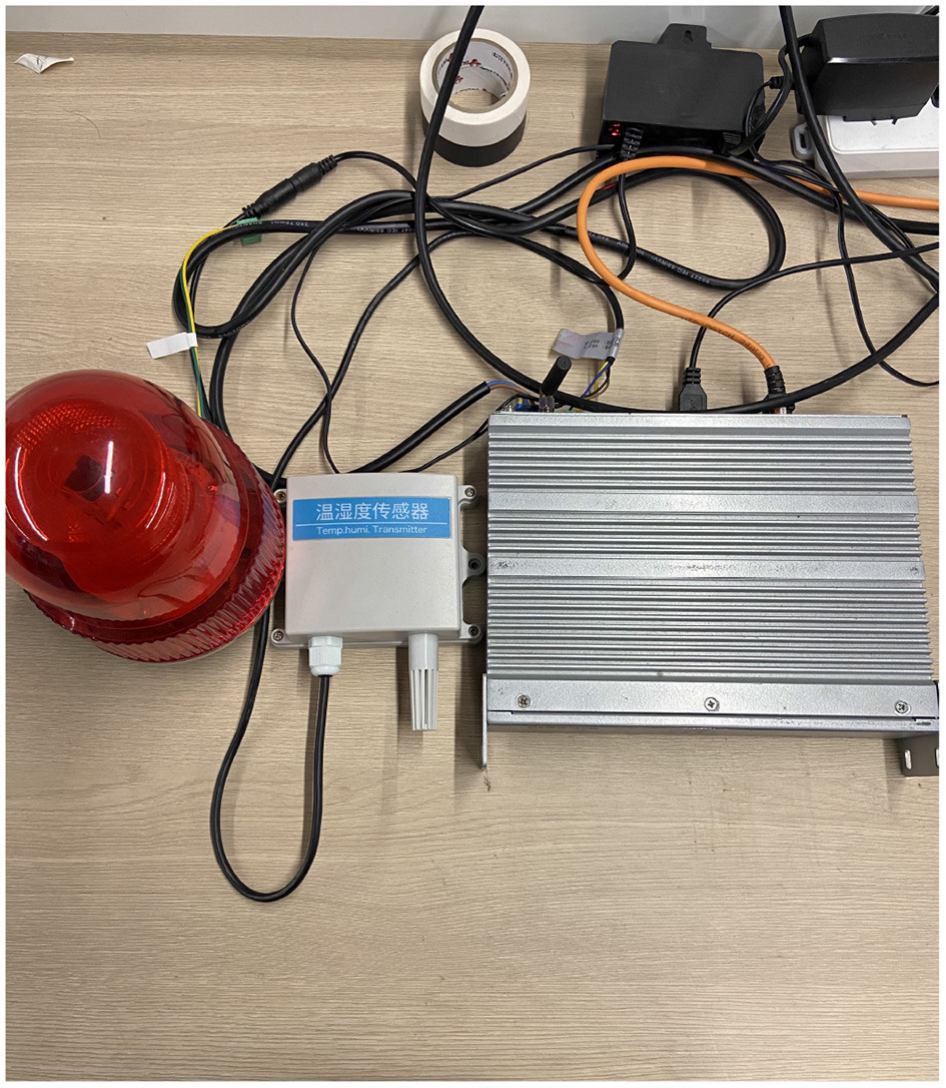
Gateway.

In the laboratory environment, we used one cloud server and ten edge devices for experiments. In the *Rinegan*, these edge-side devices receive the images or video collected by the camera, then they execute the object detection and image cropping tasks. The configurations of the CPU, memory of these devices are shown in the Table 1. Among these devices, device 1 to device 6 are virtual machine with heterogenous configurations, devices 7 to 10 are the real gateway containers of our laboratory (as shown in Figure 8). All these module on the devices are implemented based on Spring microservice architecture^4^ which exchange data through RESTFul api, i.e., the data is transferred based on HTTP. Our purpose is to simulate the performance of different operation systems with various configurations to reflect the adaptiveness of heterogeneity of our proposed system.

**Table 1:** Detail of devices used in this experiment.

**Device**	**CPU**	**Memory**
Cloud	Intel(R) Xeon(R) Gold 5218 CPU @ 2.30GHz 64cores	251G
Device1	Intel I7-9700 (8 CPUs * 1 core)	2G
Device2	Intel I7-9700 (8 CPUs * 1 core)	4G
Device3	Intel I7-9700 (8 CPUs * 1 core)	8G
Device4	Intel I7-9700 (1 CPU * 8 cores)	4G
Device5	Intel I7-9700 (1 CPU * 4 cores)	8G
Device6	Intel I7-9700 (1 CPU * 1 core)	8G
Device7	RK 3309	2G
Device8	RK 3309	2G
Device9	RK 3309	4G
Device10	RK 3309	4G

### 4.2. Performance Evaluation

We compared the running time of the two methods we mentioned above. In the *Rinegan*, the running times for face detection and image cropping on the edge devices (100 images per device) are 62, 58, 56, 59, 64, 66, and 67 s in order, respectively. The face recognition on the cloud is about 120 s. In total, the entire process consumes about 187 s. As for the *Centralized* case, it takes 230 s to process all the images in the cloud. Therefore, in our experimental environment, our architecture can increase the processing speed by about 19% (43 s less). It should be noted that in the real environment, there may be far more than 10 edge nodes. So it is foreseeable that the processing speed of our architecture will be faster also.

We also measure the video processing ability of our architecture. In this experiment, when running with 10 edge side devices (we clipping the video into 10 pieces each with 7.8 s per device), we find a abnormal result that the detection tasks takes about 3 s per frame, i.e., 3*30*7.8 = 700 s in total, while the cloud spends only about 570 s to run both recognition and detection. This result indicates that the entire resource of edge side is less than the cloud server in Table 1. Therefore, we implement 10 more edge devices with the same configurations to reexamine the performance, each processes about 3.9 s video. In total, the detection and recognition tasks take about 340(edge)+125(cloud) = 465 s, which shows a 18% improvement of efficacy. In real world, the edge side devices may be much more than cloud side and can be equipped with more computing resource if necessary. As we mentioned above, since the video processing is essentially similar to process a sequence of pictures, the increased efficacy depends on the entire computation resources of edge side devices. Moreover, the low rate of video process is caused by the higher resolution ratio rather than the difference of video and picture format. We have to notice that how to improve the efficacy of processing high quality pictures is not concerned in this work. Our purpose is to present a distributed architecture that achieves a better performance than centralized mode.

The cropping process performed on the edge side significantly reduces the size of the total pictures and video by about 45 and 90%, respectively, which therefore greatly reduces the amount of data to be transferred as well as the bandwidth consumption. Another advantage of cropping is that it can prevent the 45 and 90% data from being leaked when transferring through untrusted network channel. Specifically, the surrounding environment which may contains sensitive information can be cropped before transmission.

In summary, the *Rinegan* system we developed effectively reduces bandwidth consumption and significantly improves the execution speed. Moreover, the data leakage risk can be remarkably decreased by our cropping process. More importantly, our system has flexibility and scalability compared with the centralized image process architecture, and can be deployed more flexibly at the edge. Additionally, though the process of all frames (2,356 in total) in our video is resource intensive one can select a part of them, e.g., 1 frame per second, or compress the pictures to improve the detection efficacy.

## 5. Discussion

From the above experimental results and comparative analysis, the *Rinegan* system we designed performed well, but we also discussed and thought about this work, as shown below.

1. *Limitation of this work*. Our experiment is not large enough as real world IoT scene. However, considering our heterogeneous devices, one of which even configures 1 cpu core, we can infer that our system can have good performance in large-scale scenarios. In this work, we have not determined how to allocate tasks on the edge and cloud to maximize the performance of the system, but this does not affect the current use of the system.
2. *Prospect*. In future work, we should conduct multiple experiments to determine the optimal task volume ratio between the edge and the cloud to optimize the performance of the system. Moreover, our research found that the architecture we proposed is not only suitable for intelligent security robots, but also for the construction in the IoT large-scale scenarios (Qiu et al., 2020; Shafiq et al., 2020). For example, we can deploy this system on smart light poles to achieve collaborative work between the edge and the cloud. We have reason to believe that this will greatly improve the efficiency of smart city construction.

## 6. Conclusion

In this work, we propose a scalable architecture that can improve the image and video processing capabilities of the intelligent security robot and facilitate the tracking task. We reduce the bandwidth consumption of the cloud by deploying distributed image processing functions on the edge. At the same time, by cropping pictures, our architecture can also effectively protect privacy. We developed a system *Rinegan* with this architecture and tested the system in a laboratory environment. The result shows that *Rinegan* consumes less resources and Executes in a shorter time in cloud compared with centralized system. At the same time, our system takes into account the scalability and performs better in large-scale scenarios.

## Data Availability Statement

The original contributions presented in the study are included in the article/supplementary material, further inquiries can be directed to the corresponding author/s.

## Author Contributions

XL provided the core idea of this work and did background research. LF and YL wrote the paper and designed the thesis plan. HX and YZ designed the experimental plan and provided help for the result analysis. LY provided the research group with financial support and experimental equipments, as well as being a supportive corresponding author. All authors contributed to the article and approved the submitted version.

## Conflict of Interest

The authors declare that the research was conducted in the absence of any commercial or financial relationships that could be construed as a potential conflict of interest.

## Publisher's Note

All claims expressed in this article are solely those of the authors and do not necessarily represent those of their affiliated organizations, or those of the publisher, the editors and the reviewers. Any product that may be evaluated in this article, or claim that may be made by its manufacturer, is not guaranteed or endorsed by the publisher.

## Footnotes

^1^*Celeba*. Available online at: http://mmlab.ie.cuhk.edu.hk/projects/CelebA.html.

^2^*Face-Recognition*. Available online at: https://face-recognition.readthedocs.io/en/latest/face_recognition.html.

^3^*Face Recognition Example*. Available online at: https://github.com/ageitgey/face_recognition/tree/master/examples.

^4^*Spring Boot*. Available online at: https://spring.io/projects/spring-boot.

## References

[B1] Bevi A. R. Shakthipriya P. Malarvizhi S. (2019). Design of software defined networking gateway for the internet-of-things. Wireless Pers. Commun. 107, 1273–1287. 10.1007/s11277-019-06335-9

[B2] Bistritz I. Bambos N. (2019). Asymptotically optimal distributed gateway load-balancing for the internet of things, in 10th International Conference on Networks of the Future, NoF 2019 (Rome), 98–101.

[B3] Cai Z. Fan Q. Feris R. S. Vasconcelos N. (2016). A unified multi-scale deep convolutional neural network for fast object detection, in European Conference on Computer Vision (Amsterdam: Springer), 354–370. 10.1007/978-3-319-46493-0_22

[B4] Cha S-C. Chen J-F. Su C. Yeh K-H. (2018). A blockchain connected gateway for ble-based devices in the internet of things. IEEE Access 6, 24639–24649. 10.1109/ACCESS.2018.2799942

[B5] Chang C-C. Lee W-K. Liu Y. Goi B-M. Phan R. C.-W. (2019). Signature gateway: offloading signature generation to iot gateway accelerated by GPU. IEEE Internet Things J. 6, 4448–4461. 10.1109/JIOT.2018.2881425

[B6] Chen C. Lin M. Liu C. (2018). Edge computing gateway of the industrial internet of things using multiple collaborative microcontrollers. IEEE Network 32, 24–32. 10.1109/MNET.2018.1700146

[B7] Cheng B. Solmaz G. Cirillo F. Kovacs E. Terasawa K. Kitazawa A. (2018). Fogflow: easy programming of IoT services over cloud and edges for smart cities. IEEE Internet Things J. 5, 696–707. 10.1109/JIOT.2017.2747214

[B8] Constant N. Borthakur D. Abtahi M. Dubey H. Mankodiya K. (2017). Fog-assisted wIoT: a smart fog gateway for end-to-end analytics in wearable internet of things. arXiv preprint arXiv:1701.08680.

[B9] Dolberg L. François J. Chowdhury S. R. Ahmed R. Boutaba R. Engel T. (2016). A generic framework to support application-level flow management in software-defined networks, in IEEE NetSoft Conference and Workshops, NetSoft 2016 (Seoul: IEEE), 121–125.

[B10] Dolui K. Király C. (2018). Towards multi-container deployment on iot gateways, in IEEE Global Communications Conference, GLOBECOM 2018 (Abu Dhabi), 1–7.

[B11] Kovatsch M. Lanter M. Duquennoy S. (2012). Actinium: a restful runtime container for scriptable internet of things applications, in 3rd IEEE International Conference on the Internet of Things, IOT 2012 (Wuxi: IEEE), 135–142.

[B12] Lal M. Kumar K. Arain R. H. Maitlo A. Ruk S. A. Shaikh H. (2018). Study of face recognition techniques: a survey. Int. J. Adv. Comput. Sci. Appl. 9, 42–49.

[B13] Lee K. Kuhl S. J. Bockholt H. J. Rogers B. P. Reed D. A. (2018). A cloud-based scientific gateway for internet of things data analytics, in Proceedings of the Practice and Experience on Advanced Research Computing, PEARC 2018 (Pittsburgh, PA), 34:1–34:8. 10.1145/3219104.3219123

[B14] Li B. Sun X. Yu S. (2019). Designing of internet of things sensor based information gateway using SDN concept. Int. J. Distribut. Syst. Technol. 10, 13–24. 10.4018/IJDST.2019010102

[B15] Li H. Lin Z. Shen X. Brandt J. Hua G. (2015). A convolutional neural network cascade for face detection, in Proceedings of the IEEE Conference on Computer Vision and Pattern Recognition, 5325–5334.

[B16] Li Y. Su X. Riekki J. Kanter T. Rahmani R. (2016). A SDN-based architecture for horizontal internet of things services, in 2016 IEEE International Conference on Communications, ICC 2016 (Kuala Lumpur: IEEE), 1–7.

[B17] Luo C. Tan Z. Min G. Gan J. Shi W. Tian Z. (2020). A novel web attack detection system for internet of things via ensemble classification. IEEE Trans. Indus. Inform. 17, 5810–5818. 10.1109/TII.2020.3038761

[B18] Mendki P. (2018). Docker container based analytics at iot edge video analytics usecase, in 2018 3rd International Conference On Internet of Things: Smart Innovation and Usages (IoT-SIU), 1–4.

[B19] Meng W. Gu Z. Zhang M. Wu Z. (2017). Two-bit networks for deep learning on resource-constrained embedded devices. arXiv preprint arXiv:1701.00485.

[B20] Morabito R. (2017). Virtualization on internet of things edge devices with container technologies: a performance evaluation. IEEE Access 5, 8835–8850. 10.1109/ACCESS.2017.2704444

[B21] Morabito R. Beijar N. (2016). Enabling data processing at the network edge through lightweight virtualization technologies, in 2016 IEEE International Conference on Sensing, Communication and Networking (SECON Workshops), 1–6.

[B22] Morabito R. Farris I. Iera A. Taleb T. (2017). Evaluating performance of containerized IoT services for clustered devices at the network edge. IEEE Internet Things J. 4, 1019–1030. 10.1109/JIOT.2017.2714638

[B23] Morabito R. Petrolo R. Loscrì V. Mitton N. (2016). Enabling a lightweight edge gateway-as-a-service for the internet of things, in 7th International Conference on the Network of the Future, NOF 2016 Búzios, 1–5.

[B24] Mouradian C. Jahromi N. T. Glitho R. H. (2018). NFV and SDN-based distributed iot gateway for large-scale disaster management. IEEE Internet Things J. 5, 4119–4131. 10.1109/JIOT.2018.2867255

[B25] Nefian A. V. Hayes M. H. (1998). Hidden markov models for face recognition, in Proceedings of the 1998 IEEE International Conference on Acoustics, Speech and Signal Processing, ICASSP'98 (Seattle, WA: IEEE), 2721–2724.

[B26] Ogawa K. Kanai K. Nakamura K. Kanemitsu H. Katto J. Nakazato H. (2019). IoT device virtualization for efficient resource utilization in smart city IoT platform, in IEEE International Conference on Pervasive Computing and Communications Workshops, PerCom Workshops 2019 (Kyoto), 419–422. 10.1109/PERCOMW.2019.8730806

[B27] Qi B. Wu M. Zhang L. (2017). A DNN-based object detection system on mobile cloud computing, in 17th International Symposium on Communications and Information Technologies, ISCIT 2017 (Cairns, QLD: IEEE), 1–6.

[B28] Qiu J. Du L. Zhang D. Su S. Tian Z. (2019). Nei-tte: intelligent traffic time estimation based on fine-grained time derivation of road segments for smart city. IEEE Trans. Indus. Inform. 16, 2659–2666. 10.1109/TII.2019.2943906

[B29] Qiu J. Tian Z. Du C. Zuo Q. Su S. Fang B. (2020). A survey on access control in the age of internet of things. IEEE Internet Things J. 7, 4682–4696. 10.1109/JIOT.2020.2969326

[B30] Rahmani A. M. Gia T. N. Negash B. Anzanpour A. Azimi I. Jiang M.. (2018). Exploiting smart e-health gateways at the edge of healthcare internet-of-things: a fog computing approach. Future Gener. Comput. Syst., 78, 641–658. 10.1016/j.future.2017.02.014

[B31] Redmon J. Divvala S. Girshick R. Farhadi A. (2016). You only look once: unified, real-time object detection, in Proceedings of the IEEE Conference on Computer Vision and Pattern Recognition (Las Vegas, NV: IEEE Computer Society), 779–788. 10.1109/CVPR.2016.91

[B32] Ren J. Guo Y. Zhang D. Liu Q. Zhang Y. (2018). Distributed and efficient object detection in edge computing: challenges and solutions. IEEE Netw. 32, 137–143. 10.1109/MNET.2018.1700415

[B33] Rufino J. Alam M. Ferreira J. Rehman A. Tsang K. F. (2017). Orchestration of containerized microservices for iiot using docker, in IEEE International Conference on Industrial Technology, ICIT 2017 (Toronto, ON: IEEE), 1532–1536.

[B34] Shafiq M. Tian Z. Bashir A. K. Du X. Guizani M. (2020). CorrAUC: a malicious Bot-IoT traffic detection method in IoT network using machine learning techniques. IEEE Internet Things J. 8, 3242–3254. 10.1109/JIOT.2020.3002255

[B35] Shi X. Shan S. Kan M. Wu S. Chen X. (2018). Real-time rotation-invariant face detection with progressive calibration networks, in Proceedings of the IEEE Conference on Computer Vision and Pattern Recognition (Salt Lake City, UT: IEEE Computer Society), 2295–2303. 10.1109/CVPR.2018.00244

[B36] Tan M. Pang R. Le Q. V. (2020). Efficientdet: scalable and efficient object detection, in Proceedings of the IEEE/CVF Conference on Computer Vision and Pattern Recognition (Seattle, WA: IEEE), 10781–10790. 10.1109/CVPR42600.2020.01079

[B37] Tian Z. Luo C. Qiu J. Du X. Guizani M. (2020). A distributed deep learning system for web attack detection on edge devices. IEEE Trans. Ind. Inform. 16, 1963–1971. 10.1109/TII.2019.2938778

[B38] Tian Z. Shi W. Wang Y. Zhu C. Du X. Su S.. (2019). Real-time lateral movement detection based on evidence reasoning network for edge computing environment. IEEE Trans. Indus. Inform. 15, 4285–4294. 10.1109/TII.2019.2907754

[B39] Wang Y. Tian Z. Sun Y. Du X. Guizani N. (2020). LocJury: an IBN-based location privacy preserving scheme for IoCV. IEEE Trans. Intell. Transport. Syst. 10.1109/TITS.2020.2970610

[B40] Yaseen M. U. Anjum A. Rana O. F. Hill R. (2018). Cloud-based scalable object detection and classification in video streams. Future Gener. Comput. Syst. 80, 286–298. 10.1016/j.future.2017.02.003

